# Vertical Velocity Distribution in Open-Channel Flow with Rigid Vegetation

**DOI:** 10.1155/2014/146829

**Published:** 2014-04-16

**Authors:** Changjun Zhu, Wenlong Hao, Xiangping Chang

**Affiliations:** College of Urban Construction, Hebei University of Engineering, Handan 056038, China

## Abstract

In order to experimentally investigate the effects of rigid vegetation on the characteristics of flow, the vegetations were modeled by rigid cylindrical rod. Flow field is measured under the conditions of submerged rigid rod in flume with single layer and double layer vegetations. Experiments were performed for various spacings of the rigid rods. The vegetation models were aligned with the approaching flow in a rectangular channel. Vertical distributions of time-averaged velocity at various streamwise distances were evaluated using an acoustic Doppler velocimeter (ADV). The results indicate that, in submerged conditions, it is difficult to described velocity distribution along the entire depth using unified function. The characteristic of vertical distribution of longitudinal velocity is the presence of inflection. Under the inflection, the line is convex and groove above inflection. The interaction of high and low momentum fluids causes the flow to fold and creates strong vortices within each mixing layer. Understanding the flow phenomena in the area surrounding the tall vegetation, especially in the downstream region, is very important when modeling or studying the riparian environment. ADV measures of rigid vegetation distribution of the flow velocity field can give people a new understanding.

## 1. Introduction 

Vegetation plays an important role in altering flow characteristics (such as velocity distribution, Reynolds number, and Manning coefficient) compared with nonvegetated conditions in rivers [1]. Generally, the vertical velocity distribution is related directly to the bed shear stress for nonvegetation flow, while, for vegetated flow, it is mainly decided by the vegetation drag since the vegetation roughness is much larger than river bed roughness [2]. The influence mechanism of aquatic plant on flow is very complicated, which is dependent not only on the cross-sectional shape of river, water depth, discharge but also on the species, bending rigidity, distribution, shape of vegetation, and whether it is submerged [3].

The mechanical effects of rivers with vegetation have been of primary interest for decades, because of their role in environmental fluid mechanics. A number of studies on the flow and turbulence characteristics through emergent vegetation are available [4–6]. Flow phenomena become more complicated when the flow depth exceeds the height of vegetation. Laboratory studies on fully developed flows with submerged vegetation have demonstrated reduced velocities within the vegetation zone [7, 8].

A series of laboratory experiments were performed to investigate the connection between submerged vegetation and the key geomorphology around the vegetation. The submerged vegetation was modeled as bundled plastic fibers with a variation in vegetation density. The objective of this work is to describe the detailed characteristics of flow through a simulated array of rigid vegetation by examining its effects on the velocity and observing the influence of vegetation density and heights. Measurements of velocity are taken along verticals at multiple locations at different sections to adequately represent the conditions everywhere within the flow and to capture the flow response as it moves through the vegetation array. The experiments include single layer double layer experiments. And the results are compared.

## 2. Experimental Conditions

**Figure 1:**
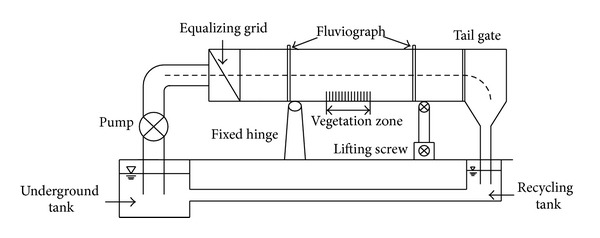
The experimental recirculating open-channel rectangle flume setup.

The experimental systems (Figure 1) were composed of two pumps to force water through the system and maintain recirculation, an inlet section with turning valves at the upstream end to control the flow discharge and generate fully developed turbulent flow, and an outlet section with a triangular adjustable weir at the downstream end to control the water level.

The flume was 7.0 m long, 0.5 m wide, and 0.8 m high with glass-sidewalls and a concrete bottom, so that the interactions between the vegetation and flow could be observed clearly. The water levels were constant at some flow rate. The vertical velocities of fully developed flow with rigid vegetation were measured.

**Figure 2:**
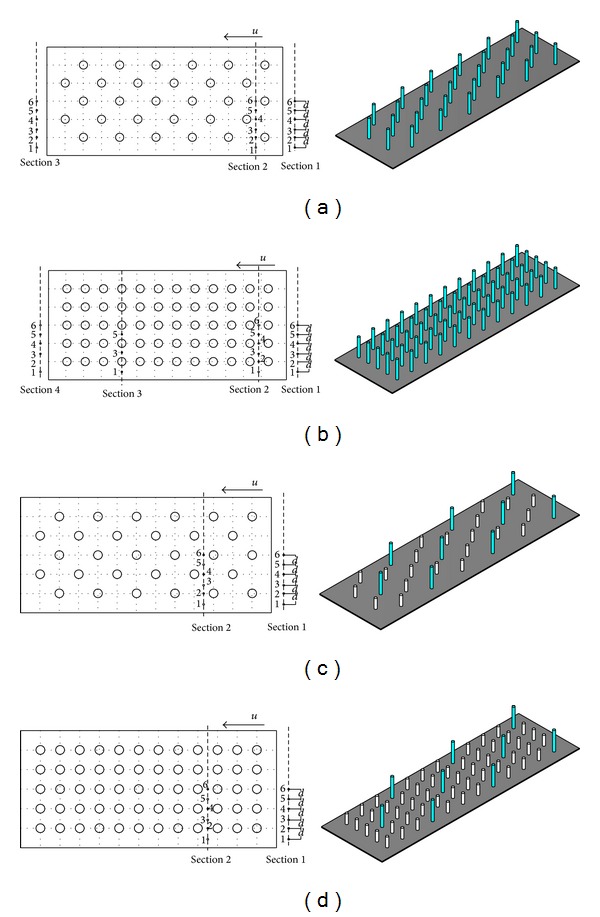
Cylindrical rod configurations and measumental locations.

The height of the rigid rod used in the arrangements (a) and (b) was 8 mm, and the heights in arrangements (c) and (d) were 4 mm and 8 mm arranged in staggered configurations (Figure 2). These experiments focused on the effects of density and height of rigid rod on the flow velocity. Measurement locations, shown in Figure 2, were chosen downstream and upstream of rigid rod. Flow characteristics were measured at three sections, numbered Sections  1 to 3 in Figure 2. Sections  1 and 3, located outside of the vegetation area, were intended for studying the effect of rising and lowering of water level on flow characteristics. Section 2 was intended for investigating the influence of rigid cyclical rod on flow characteristics. At each section, velocity was measured at six points starting from bottom of the bed to the water surface. However, due to ADV limitations and flume channel structure, the ADV sensor suspended at 5 cm above the flume bed still measures the flow characteristics at the near bed. The measurement was done at 0.5 cm increments from the bed towards the free water surface.

## 3. Results

### 3.1. Velocity Profile

**Figure 3:**
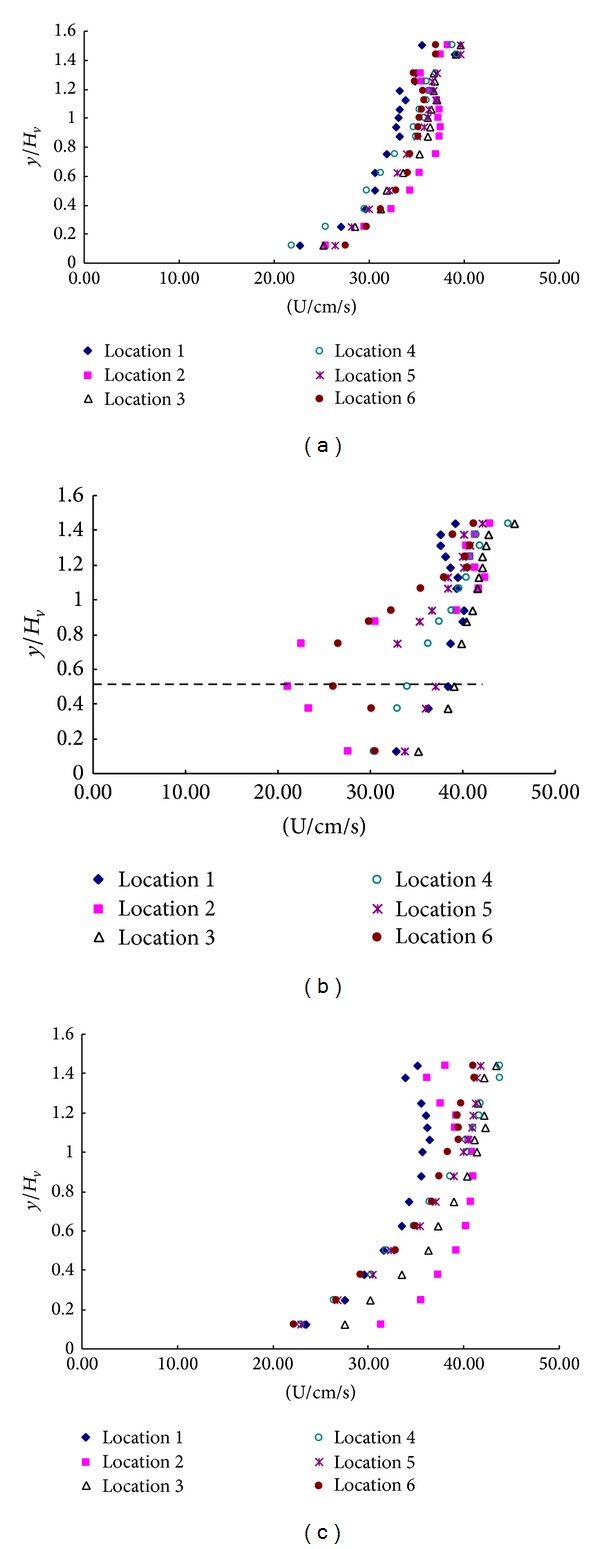
Velocity profiles of (a) upstream, (b) vegetation, and (c) downstream in the configuration a.

The vertical distribution of velocity with rigid vegetation could be divided into two layers: the upper nonvegetated layer (>1.0 Hv) and the lower rigid vegetated layer (0-1.0 Hv). The upper nonvegetated layer is from the rigid rod top (1.0 Hv) to the water surface. The lower layer is from the bed to water surface. Velocity profiles with the rigid vegetation in the first arrangement submerged are shown in Figures 3(a)–3(c).

Velocity profiles with the submerged single layer are shown in Figures 3(a)–3(c). The velocity in vegetations is different from the velocities upstream and downstream of vegetation which is complied with the semilogarithmic formula. As seen from Figure 3(b) in vegetation region, in the back of the rigid rod, the velocity is slower (location 2 and location 6) and the slowest velocities approximately are at *y*/*H*_*v*_ = 0.5. This is similar to the velocity profile immediately behind a dowel for submerged vegetation flow shown at location 2 and location 6 in Figure 3(b) and that of other researchers (e.g., Shimizu and Tsujimoto, 1994; Lopez and Garcia, 2001; and Righetti and Armanini, 2002).

**Figure 4:**
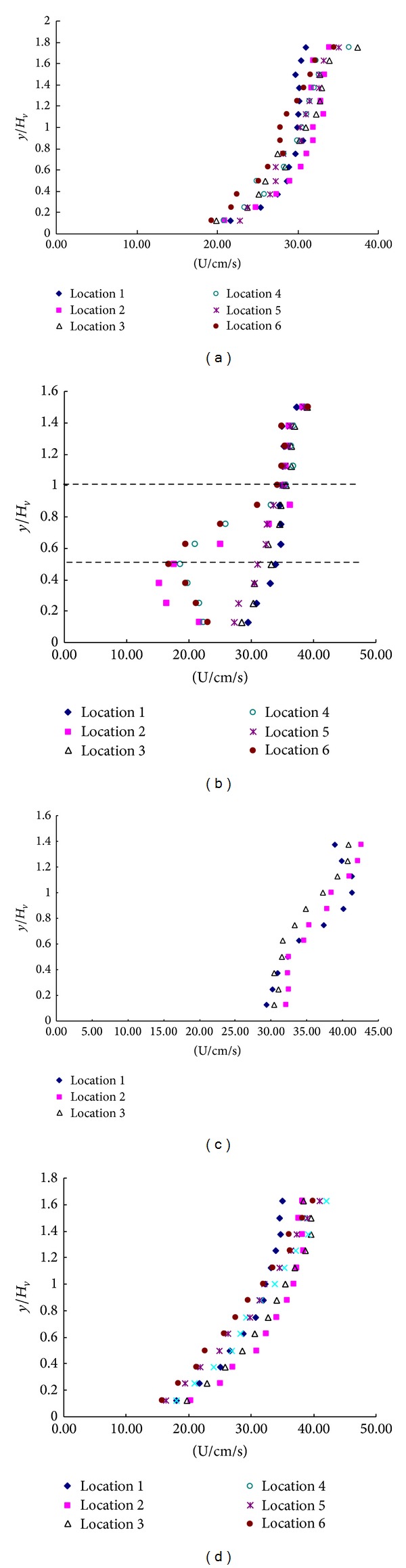
Velocity profiles of (a) upstream, (b) vegetation, and (c) downstream in the configuration b.

Velocity profiles with the submerged single layer are shown in Figures 4(a)–4(d). This is similar to the velocity in configuration a (Figure 3). As seen from Figure 4(b) in vegetation region, in the back of the rigid rod, the velocity is slower (location 2, location 4, and location 6), the slowest velocities approximately are *y*/*H*_*v*_ = 0.5, and the shape of the velocity is “*S*,” while, in the upstream and the downstream, the velocity complies with the semilogarithmic formula. But because of the influence of the vegetation, in the downstream region, the lowest velocity is in the middle of the flume (location 6); the fastest velocity is in location 2 and location 3, which can be seen in Figure 4(d).

**Figure 5:**
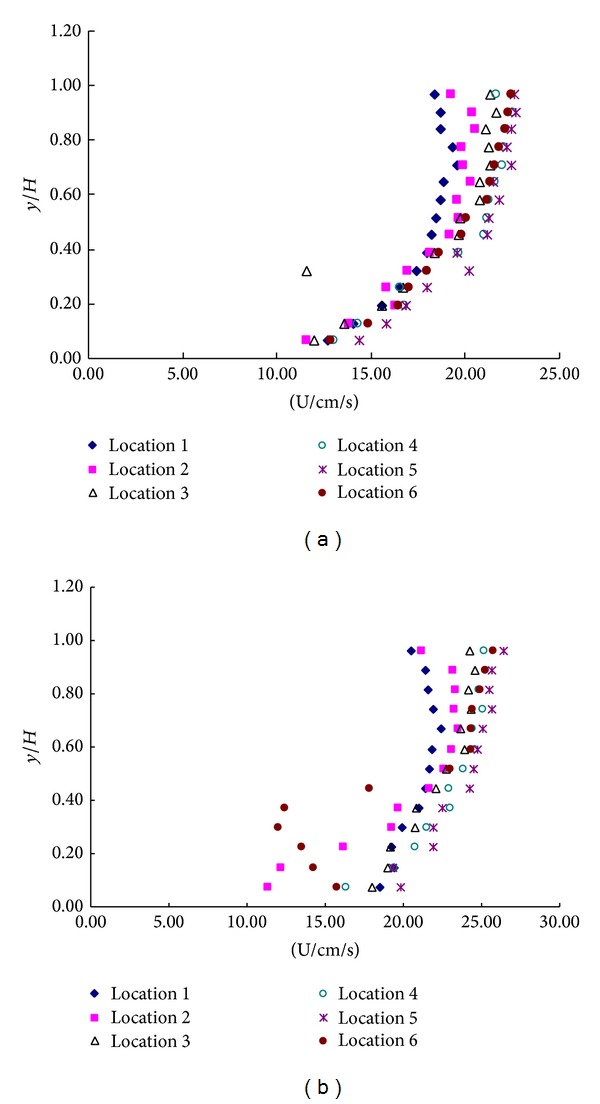
Velocity profiles of (a) upstream and (b) vegetation in the configuration c.

**Figure 6:**
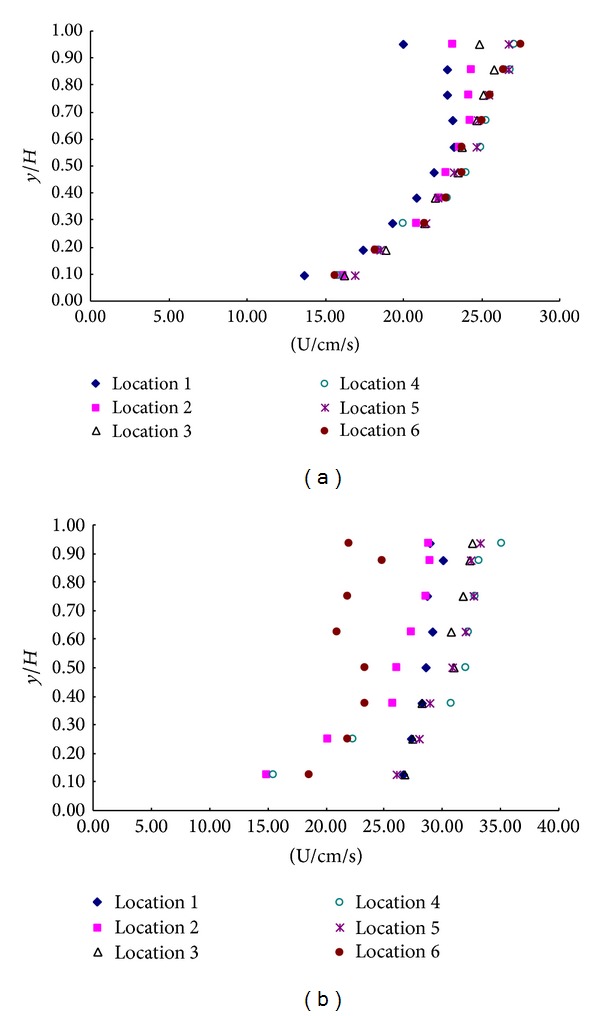
Velocity profiles of (a) upstream and (b) vegetation in the configuration d.

Velocity profiles with the short rigid rod submerged are shown in Figure 5 and Figure 6. As seen from Figure 6, in double layer flows, the fastest velocities are in the lower free stream region (locations 4) and the slowest velocities are in the region immediately downstream of a tall rigid rod (locations 6). The velocities just beside a rigid rod (locations 1, 3, and 5) are approximately midway between the fastest and slowest velocities. These results are consistent with observations made from the submerged single layer experiment (Figure 3 and Figure 4).

## 4. Conclusions

In this paper, flows through double layer vegetation and single layer vegetation are simulated in flume. The flows in double layer vegetation are more complex than in the single layer case. Some conclusions can be drawn as follows.

(1) The existence of submerged plants increases the flow gradient, and the roughness of the bed surface becomes larger, which plays the role of spoiler, and with increasing plant density is more obvious.

(2) With the increasing of plant density, backwater phenomenon exists and is significant. But with the increasing of water depth, the phenomenon is weakened. Therefore, the calculation of flow over the river containing plant must consider the impact of plant resistance.

(3) In submerged conditions, it is difficult to describe velocity distribution along the entire depth using unified function. The characteristic of vertical distribution of longitudinal velocity is the presence of inflection. Under the inflection, the line is convex and groove above inflection.

(4) The interaction of high and low momentum fluids causes the flow to fold and creates strong vortices within each mixing layer. Understanding the flow phenomena in the area surrounding the tall vegetation, especially in the downstream region, is very important when modeling or studying the riparian environment.
